# Speckle Noise Filtering in Side-Scan Sonar Images Based on the Tucker Tensor Decomposition

**DOI:** 10.3390/s19132903

**Published:** 2019-06-30

**Authors:** Jakub Grabek, Bogusław Cyganek

**Affiliations:** Department of Computer Science, Electronics and Telecommunications, AGH University of Science and Technology, Al. Mickiewicza 30, 30-059 Kraków, Poland

**Keywords:** speckle noise filtering, side-scan sonar image enhancement, Tucker tensor decomposition

## Abstract

Real signals are usually contaminated with various types of noise. This phenomenon has a negative impact on the operation of systems that rely on signals processing. In this paper, we propose a tensor-based method for speckle noise reduction in the side-scan sonar images. The method is based on the Tucker decomposition with automatically determined ranks of factoring tensors. As verified experimentally, the proposed method shows very good results, outperforming other types of speckle-noise filters.

## 1. Introduction

Side-scan sonar allows for underwater object detection thanks to the measurement of the sent and reflected low-frequency waves. This way, obtained sonar images can be processed for object recognition, obstacle avoidance, and drone manoeuvring, to name a few. However, sonar images are heavily contaminated with unwanted signals with the most prominent being speckle noise. This type of noise arises from the coherent interference of backscattered waves due to the physical properties of the object surfaces as well as wave propagation. In this paper, we tackle the problem of sonar image filtering, which allows for noise removal while preserving useful information stored in the original signals. For this purpose, the tensor-based filtering method is proposed, which accounts well for the multi-dimensionality of the sonar images. To the best of our knowledge, this is the first work in which the Tucker-based tensor decomposition with automatic rank determination is proposed for filtering of the side-scan sonar images. The obtained results show that the proposed method outperforms many other filtering methods [1,2,3].

The rest of the paper is organized as follows. Section 2 describes the related works. Section 3 briefly highlights the physical properties of the speckle noise, its model, as well as various filter types for its reduction. Our proposed tensor-based denoising method is explained in Section 4. Experimental results with a discussion on results are described in Section 5. Finally, Section 6 concludes the paper.

## 2. Related Works

Speckle noise is an unwanted phenomenon encountered in many electronic systems. In this section, we provide an overview of the known methods that were proposed for filtering of this type of signal distortion. Many papers address the speckle noise problem encountered in the domains of medical ultrasound, radars and optical coherence tomography (OCT), often proposing state-of-the-art solutions to the problem [2,3,4,5].

The speckle noise reduction is a well-defined problem, and many methods have been developed to achieve a stable balance between the level of filtering and detail preservation. Apart from the well-known simple filters, the hybrid and wavelet filters have gained attention from the researchers. In this respect, the system proposed by Yu and Acton employs a nonlinear anisotropic diffusion technique (SRAD) for removing multiplicative noise in imagery [4]. They used a partial differential equation approach which, due to the filter size and shape, allows for the generation of an image scale space without bias. SRAD, as an adaptive technique, not only preserves edges but also enhances them by inhibiting diffusion across edges.

Karthikeyan and Chandrasekar proposed a method combining the SRAD filter with the wavelet-based BayesShrink technique [5]. An input image is decomposed using the Discrete Wavelet Transform, followed by a nonlinear thresholding step. The data are then reconstructed by the Inverse Discrete Wavelet Transform. Their method achieved a PSNR metric value of over 70 dB, as measured on a test dataset.

On the other hand, in the paper by Vanithamani et al. [1], the Modified Hybrid Median Filter is presented. The method is a three-step ranking operation using a non-standard window. The system uses neighbors forming “X” and “+” shapes, overcoming the problem of edge degradation.

A system for speckle reduction for the OCT images was proposed by Adabi et al. [2]. It is based on a multi-layer perceptron neural network, which estimates parameters for the filtering module of the system. As reported, the achieved average parameter estimation is above 99%. The denoising, based on the Rayleigh distribution model, is conducted independently on the small blocks generated from the image. The final output is combined into one image, with simultaneous block artifacts reduction.

However, neither of the above solutions accounts for the multi-dimensionality of the input signal. A method proposed in this paper, which relies on signal representation and processing with tensors, accounts for this effect what leads to superior results, as discussed below.

## 3. Characteristics of the Speckle Noise and Its Filtering Methods

Speckle noise is one of the primary sources of visual noise in sonar, ultrasound or radar images [6,7]. It is mainly caused by the returning wave interference inside the transducer due to the roughness of the material surface in the wavelength scale. The scattered signal adds coherently producing patterns of constructive and destructive interference, visible as brighter or darker dots in an image. Speckle noise can be modeled as follows.

(1) g(m,n)=f(m,n)u(m,n)+η(m,n)

where g(m,n) denotes corrupted image matrix at spatial position (m,n); u(m,n) and η(m,n) stand for the multiplicative and additive component of of the noise, respectively; and *f* is the original image.

Speckle noise filtration is mainly based on assumptions that the signal and the noise are statistically independent, and the sample mean and variance of a single pixel are equal to the mean and variance of the whole local area. The different methods developed to eliminate speckle noise can be divided into adaptive and non-adaptive ones. In theory, they are low pass filters removing high-frequency noise. However, the negative side effect of this process is degradation of useful image features such as edges, corners, and other high-frequency patterns [8]. The common filtering methods, as well as their properties, are outlined in the following sections.

### 3.1. Average Filter

The average filter calculates the grey level from the mean of all pixels in a kernel surrounding the center of the window and returns a value of this central position. The mean for m×n window region is determined as follows:

(2) $$I\left(x,y\right)=\frac{1}{mn}\sum_{k\in \{m\}}\sum_{l\in \{n\}}g\left(k,l\right)$$

where *I* denotes pixel intensity of an m×n patch, *g* is intensity of a noisy pixel, at grid coordinates {m}×{n} around the central pixel position (x,y).

### 3.2. Median Filter

In the median filter, the grey level for actual window position is calculated from the median value of all pixels in the surrounding kernel of m×n. Similar to the average filter, median filtering smooths the image reducing also noise. However, due to its nonlinearity, the median filter has better performance in edge preservation and impulse noise removal than the average filter. On the other hand, the median filter has higher computational cost as a result of a need for sorting values of at least half of the pixels in the kernel. The median filtering algorithm can be outlined as follows [9]:

1. Take an m×n kernel centered around a pixel (x,y).
2. Sort the intensity values of the pixels in the kernel into ascending order.
3. Select the middle value as the new value for the pixel (x,y).

### 3.3. Frost Filter

The Frost filter calculates the grey level for each pixel using an exponentially dumped convolution kernel that can adapt its parameters [4]. This is kind of a guided filter in which its local adjustment level is controlled by the adaptive filter coefficient calculated for each window.

The filter smooths image data without removing edges or sharp features in the images while minimizing the loss of valuable information. In homogeneous areas, speckles are removed using a low-pass filter. On the other hand, in the areas containing isolated point targets, the filter preserves the observed value. More precisely, the Frost filter can be defined as follows

(3) $$I_{s}=\sum_{p\in \eta_{s}}m_{p}I_{p}$$

(4) $$m_{p}=exp\left(-KC_{s}^{2}d_{s,p}\right)/\sum exp\left(-KC_{s}^{2}d_{s,p}\right)$$

(5) $$d_{s,p}=\sqrt{{\left(x-x_{p}\right)}^{2}+{\left(y-y_{p}\right)}^{2})}$$

where *K* denotes dumping factor and $I_{s}$ is an intensity value of the center pixel in the kernel. Values (x,y) and $\left(x_{p},y_{p}\right)$ indicate grid coordinates of the centre of the window and the pixel *p*, respectively. $C_{s}$ is a local statistic value used for adaptive computation of the filter coefficients.

### 3.4. Lee Filter

The Lee filter is another adaptive method based on calculation of a local statistics [10]. A low value of the variance of the kernel causes the algorithm to have a lower impact on the image as a low pass filter. This allows for detail preservation in both low and high contrast images. Formally, the Lee filter is defined as follows

(6) $$I_{s}\left(i,j\right)=I_{m}+W\left(I_{cp}-I_{m}\right)$$

where $I_{m}$ and $I_{cp}$ denote mean intensity value of the kernel window and a value for the center pixel, respectively. The size of its filter window *W* is given as follows

(7) $$W=\sigma^{2}\left(\sigma^{2}+\rho^{2}\right)$$

where $\sigma^{2}$ and $\rho^{2}$ are variances computed for an image and estimated for the additive noise, respectively.

### 3.5. Kuan Filter

The Kuan filter transforms the multiplicative noise model into an additive noise model and then applies the local linear minimum mean square error criteria [11]. This method is similar to the Lee filter except that the weighting function is defined as follows

(8) $$W=\frac{\left(1-C_{u}/C_{i}\right)}{\left(1+C_{u}\right)}$$

where $C_{u}$ denotes an estimated noise variance and $C_{i}$ is a variance of an image.

### 3.6. Enhanced Lee Filter

The Enhanced Lee method calculates the grey level for each pixel similar to the already described Lee filter but additionally using two threshold values [12]. These thresholds control the allowable local variance and determine filter performance. Namely, low pass filtering is active when the local variance coefficient is below a lower threshold. On the other hand, the all pass filter is applied when the coefficient is above the higher threshold. For the coefficient value between the thresholds, the output is calculated with a weighting function balancing between averaging and the identity operations, respectively.

## 4. Tensor-Based Speckle Noise Filtering

Tensors are mathematical objects which can be regarded as multidimensional arrays of data, in which each separate dimension corresponds to a different degree of freedom of a measurement. Such an approach provides tools which extend the classical matrix analysis, and which can take into account correlations hidden in data, to yield better results in various applications, such as filtering [9].

### 4.1. Multi-Dimensional Signals Filtering in the Tensor Framework

Tensors extend the notion of vectors and matrices into higher-dimensional objects [9,13,14,15]. As discussed below, they allow for better representation and processing of the multidimensional signals and, in effect, also for better filtering.

A multidimensional measurement T can be expressed as a sum of pure signal tensor S and noise N caused by imperfections introduced during the measurement process as well as by other physical phenomena [15]. Hence,

(9) T=S+N

where $T,S,N\in {ℜ}^{N_{1}\times N_{2}\times \ldots \times N_{P}}$ are *P*th order tensors. The multidimensional filtering can based on the following tensor product

(10) $$\hat{T}=T\times_{1}F_{1}\times_{2}F_{2}\ldots \times_{P}F_{P}$$

where filtered version of noisy tensor T is denoted as $\hat{T}$, whereas $F_{i}$ is the *i*th mode filtering matrix. In the above equation, the *k*th modal product $T\times_{k}M$ of a tensor $T\in {ℜ}^{N_{1}\times N_{2}\times \ldots \times N_{P}}$ and a matrix $M\in {ℜ}^{Q\times N_{k}}$ is used. The result is also a tensor $S\in {ℜ}^{N_{1}\times N_{2}\times \ldots N_{k-1}\times Q\times N_{k+1}\times \ldots \times N_{P}}$, whose elements are expressed as follows:

(11) $$S_{n_{1}n_{2}\ldots n_{k-1}qn_{k+1}\ldots n_{P}}={\left(T\times_{k}M\right)}_{n_{1}n_{2}\ldots n_{k-1}qn_{k+1}\ldots n_{P}}=\sum_{n_{k}=1}^{N_{k}}t_{n_{1}n_{2}\ldots n_{k-1}qn_{k+1}\ldots n_{P}}m_{qn_{k}}$$

As shown below, the filter matrices $F_{i}$, called factors, can be obtained using the Tucker decomposition of tensors [16]. Thanks to the proper selection of the ranks of the tensor decomposition factors, decomposition usually well separates useful signal from noise, at the same time taking multidimensional characteristics of the signal into account. The decomposition procedure of the tensor T is done by calculation of an approximating tensor $\hat{T}$ that is close to the input tensor in terms of the Frobenius norm. Hence, a minimization function is defined as follows

(12) $$\Theta \left(\hat{T}\right)=\left|\right|\hat{T}-T{\left|\right|}_{F}^{2}$$

**Figure 1:**
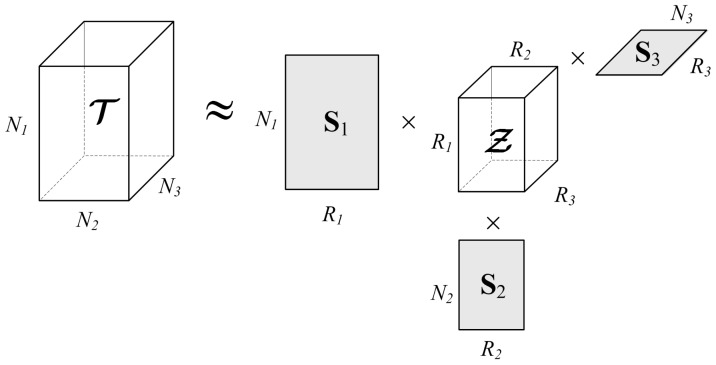
Visualization of the Tucker decomposition of a 3D tensor.

The concept of the Tucker decomposition of a 3D tensor is presented in Figure 1. Assuming that the approximating tensor $\hat{T}$ contains the same amount of useful information as the original tensor T, it can be expressed as follows

(13) $$\hat{T}=Z\times_{1}S_{1}\times_{2}S_{2}\times_{3}\ldots \times_{P}S_{P}$$

where $Z\in {ℜ}^{R_{1}\times R_{2}\times \ldots \times R_{P}}$ is a core tensor and $S_{i}\in {ℜ}^{N_{i}\times R_{i}}$ are the so-called mode matrices. Using algebraic operations, from Equation (13), the formula for the core tensor is obtained:

(14) $$Z=\hat{T}\times_{1}S_{1}^{T}\times_{2}S_{2}^{T}\times_{2}\ldots \times_{P}S_{P}^{T}$$

Combining Equation (14) with Equations (12) and (13) yields

(15) $$\Theta \left(\hat{T}\right)=\left|\right|\hat{T}-T\prod_{k=1}^{P}\times_{k}\left(S_{k}S_{k}^{T}\right){\left|\right|}_{F}^{2}$$

The Tucker decomposition in Equation (15) reads that a tensor T is approximated by its projection onto space spanned by the matrices $S_{k}$. To compute the series of $S_{k}$ matrices, the alternating method can be used [9,17,18,19].

Finally, it can be shown that the factor matrices $F_{i}$ can be obtained from the tensor decomposition matrices $S_{i}$, as follows [15]

(16) $$F_{i}=S_{i}S_{i}^{T}\;$$

The approximation in Equation (13) includes only the components conveying the majority of energy available in the signal. However, to ensure the best quality, the estimation of the proper ranks $R_{1}$, $R_{2}$, and $R_{3}$ of the mode matrices $S_{i}$ is necessary. Although fixed values can be used as a first approximation, in real dynamic systems with unpredictable noise value, the proper ranks need to be based on the signal content. To solve this problem, the method presented by Muti and Bourennane [14] is used. For this purpose, the minimum description length parameter (MDL) is computed for each dimension separately. For an argument *i*, $1\leq i\leq N_{k}$, a value of MDL is given as follows

(17) $$MDL\left(i\right)=-\log\frac{\prod_{j=i+1}^{N_{k}}\lambda_{j}^{1/(N_{k}-i)}}{\left[1/N_{k}-i\right]\sum_{j=i+1}^{N_{k}}\lambda_{j}}+\frac{1}{2}i\left(2N_{k}-i\right)\log c_{k}$$

where $\lambda_{j}$ denotes *j*th eigenvalue in the eigendecomposition and $c_{k}$ is a number of observations. Such an approach is also used in the presented filtering system.

### 4.2. The Tensor Filtering Algorithm

The Tucker tensor decomposition algorithm consecutively computes the dominating eigenspace spanned by the flattened tensor at each tensor dimension [9]. In each such a step, a minimum $i_{min}$ is sought as a last component number from eigenvalues with significant decomposition impact.

The proposed method utilizes a moving window pattern, which is common in image processing [6]. The kernel of size of $w_{s}\times w_{s}$ traverses image *I* from the upper-left to the bottom-right corner, respectively, with the step fixed as $w_{s}/4$ to ensure area overlapping for better representation. Such movement of the kernel results in a number of patches that are collected into the input tensor T. Depending on the Close Neighbor Distance (CND) parameter, more or fewer windows from the close neighborhood are included. For CND=1, which is the lowest possible value, there are nine windows combined into the input tensor. This procedure is outlined in Algorithm 1.

This way, the prepared tensor is Tucker decomposed with the help of the Higher Order Orthogonal Iteration (HOOI) algorithm [16]. In the result, the core tensor and the set of mode matrices are obtained. These, in the reverse signal synthesis process, are then used for signal filtering, as described in the previous section. This way approximated signal contains most important information with noise significantly attenuated due to properly adjusted rank of the tensor decomposition. In the next step, this way filtered signal patch is inserted to the output image *X* into a position corresponding to the actual position of the kernel in *I*. The process repeats itself until the end position is reached. Then each pixel in *X* is scaled by dividing its value by a number of its occurrences during the filtering process. The output image is then returned. The routine is implemented as shown in Algorithm 2. As alluded to previously, to determine proper ranks of the tensor, the MDL calculation process, as in Equation (17), is performed for all tensor dimensions.

**Algorithm 1** Tensor assembler.
 1:**procedure**generate tensor(*I*, *x*, *y*, $w_{s}$, CND) 2:  T=∅ 3:  **for** m=x−CND:x+CND **do** 4:   **for** n=y−CND:y+CND **do** 5:     crop a rectangular patch with a top-left corner point at *n*, *m* and size $w_{s}$ 6:     append window to *T* 7:   **end for** 8:  **end for** 9:  **return** *T*10:
**end procedure**



**Algorithm 2** Filtering algorithm.
 1:**procedure**filter tucker(*I*, $t_{r}$, $w_{s}$, CND) 2:  form a zero filled initial output image *X* of the same dimensions as *I* 3:  **for** $m=1:w_{s}/4:M$ **do** 4:   **for** $n=1:w_{s}/4:N$ **do** 5:     GENERATE TENSOR(*I*, *m*, *n*, $w_{s}$, CND)         ▹ generate filtering tensor 6:     **if** $t_{r}=\emptyset$ **then** 7:      **for** k=1:3 **do** 8:        calculate $t_{r}\left[k\right]$ for *k*-axis using MDL 9:      **end for**10:     **end if**11:     reconstruct window *Y* with Tucker reconstruction and the ranks $t_{r}$12:     add window *Y* to *X*13:   **end for**14:  **end for**15:  rescale window *X*16:  **return** *X*17:
**end procedure**



## 5. Experimental Results

The presented method was implemented in Python, using the numpy, scipy and skimage libraries. Additionally, for tensor decomposition, the TensorLy library was used [20]. The benchmark methods were implemented using the PyRadar library with small changes, allowing them to be run in the test environment. Experiments presented in this section were performed on a laptop computer equipped with 16 GM of RAM, 6-core processor i7-8750H with the 2.2 GHz base clock, and 64-bit Ubuntu 18.04 OS.

However, finding proper test sonar images is difficult. Therefore, for the quantitative evaluation, the synthetic sonar images were generated, which contain different types of objects. During the experiment, the set of five images was contaminated by the addition of speckle noise generated as follows

**Figure 2:**
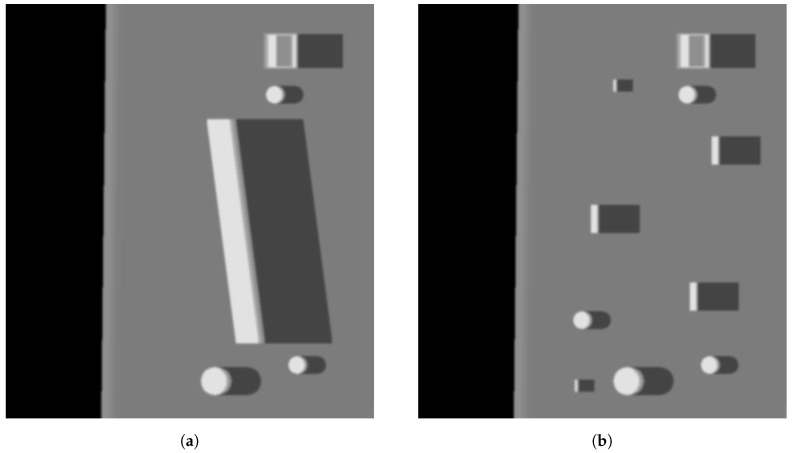
Exemplary synthetic images used as an input. (**a**) Sample image containing larger generated objects; (**b**) Sample image containing smaller generated objects.

**Figure 3:**
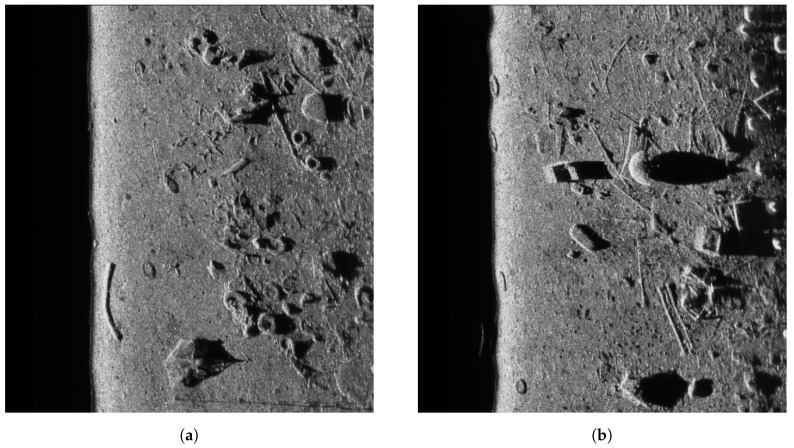
Exemplary real side scan sonar images used as an input. (**a**) Sample image containing man-made circular objects (tyres); (**b**) Sample image containing man-made circular and box-shaped objects.

(18) g=f+n·f

where *n* is a uniform noise with the mean $\bar{x}=0$ and variance $\sigma^{2}$ = 0.0001, 0.0005, 0.001, 0.005, 0.01, 0.05, and 0.1. Matrices *g* and *f* denote corrupted image and original image, respectively. For better demonstration of filtering capabilities of all methods, the real sonar images were also used. Examples are presented in Figure 2 and Figure 3 for the synthetic and and real side scan sonar images, respectively.

The full code, as well as all generated images and plots, are available in an online repository [21]. The quantitative results were measured in terms of the Peak Signal to Noise Ratio (PSNR), Structural Similarity (SSIM), and Mean Squared Error (MSE) parameters calculated for each picture, respectively [22,23]. The MSE for an n×m image *I* and its noised approximation *K* is defined as follows:

(19) $$MSE=\frac{1}{mn}\sum_{i=0}^{m-1}\sum_{j=0}^{n-1}{\left[I\left(i,j\right)-K\left(i,j\right)\right]}^{2}$$

On the other hand, PSNR is a term for the ratio between a maximum possible pixel value $I_{max}$ and the value MSE of a noise corrupted signal. It is defined as:

(20) $$PSNR=10\log_{10}\left(\frac{I_{max}^{2}}{MSE}\right)$$

Finally, the SSIM denotes a way for a quality assessment based on the degradation of the structural information between two signals *x* and *y*, respectively, and is defined as follows:

(21) $$SSIM\left(x,y\right)=\frac{\left(2\mu_{x}\mu_{y}+C_{1}\right)\left(2\sigma_{xy}+C_{2}\right)}{\left(\mu_{x}^{2}+\mu_{y}^{2}+C_{1}\right)\left(\sigma_{x}^{2}+\sigma_{y}^{2}+C_{2}\right)}$$

where μ represents an average and $\sigma^{2}$ the variance of a given image. $\sigma_{xy}$ is the covariance between *x* and *y*, and $C_{1}$ and $C_{2}$ are constants stabilising the division with small factors in the denominator.

Edges of the objects were visually inspected to determine the impact of a filtering algorithm on their sharpness. To provide a benchmark for the proposed algorithm, the filters such as Mean, Median, Frost, Lee, Lee enhanced and Kuan were used. The window size and other input parameters for the benchmark filters were set to achieve a balance between speckle reduction and edge preservation.

**Figure 4:**
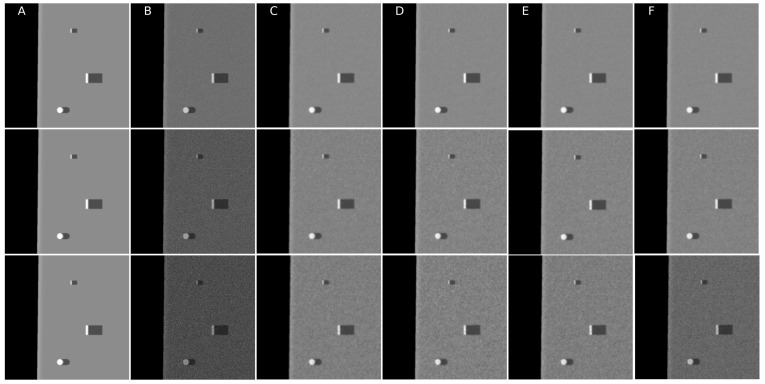

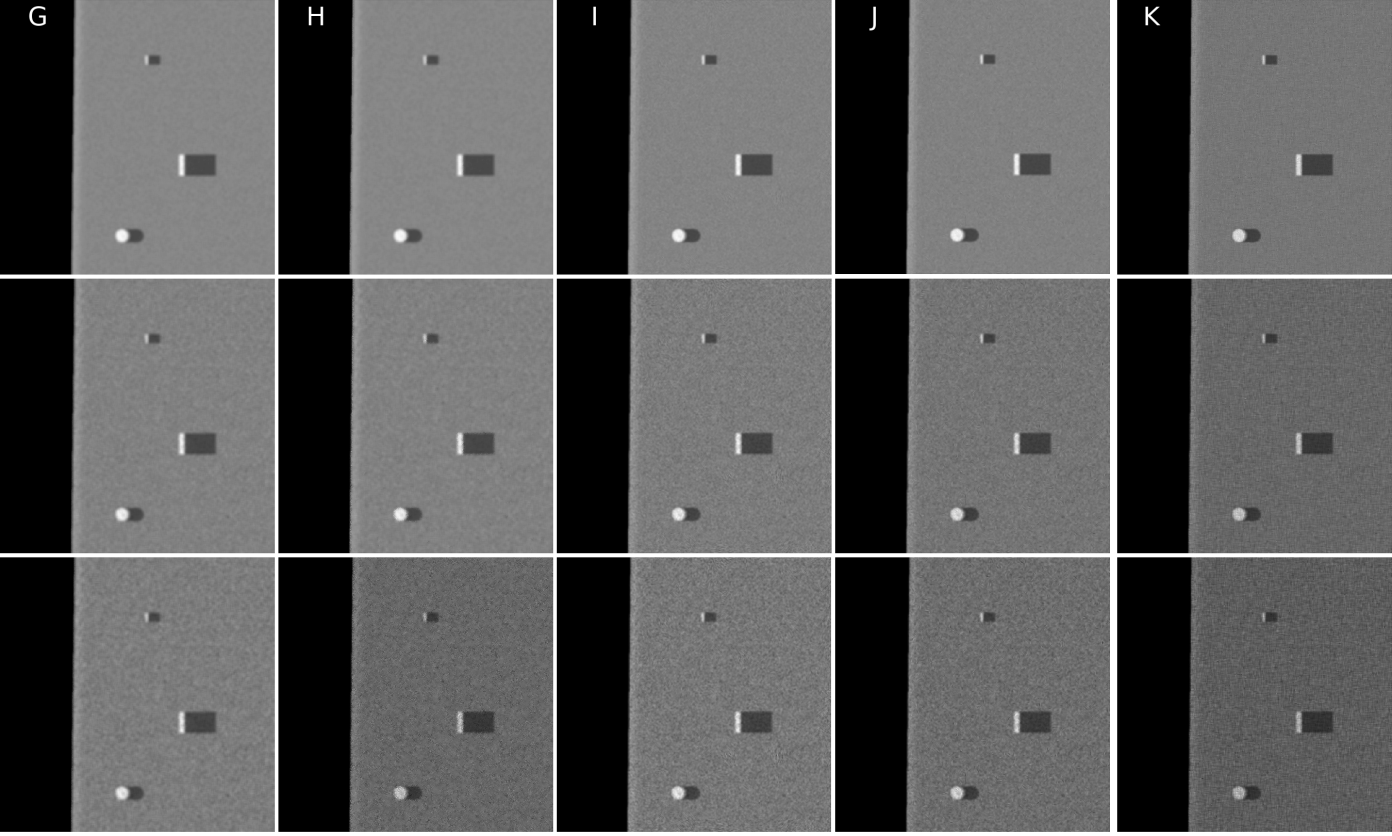
Filtering results comparison for different noise values. Top row $\sigma^{2}=0.01$, middle row $\sigma^{2}=0.05$, bottom row $\sigma^{2}=0.1$: (**A**) Original image; (**B**) noised image; (**C**) Mean; (**D**) Median; (**E**) Frost; (**F**) Lee; (**G**) Lee enhanced; (**H**) Kuan; (**I**) P1; (**J**) P2; and (**K**) PAuto.

In the presented experiments, the noise reduction, as well as the influence of various parameters on this process, were measured. This way, the obtained performance of the different denoising methods is presented in Table 1. P1 and P2 denote results for the proposed method with different values of the input parameters. Their exact values, for each P1 and P2, respectively, are presented in Table 2. The next proposed method, called PAuto, is the one endowed with the MDL algorithm for the automatic rank calculation. In this case, for every assembled tensor, a new set of ranks is estimated. The average execution time for each algorithm is presented in Table 3. On the other hand, the comparative results for all tested algorithms are collected in Figure 4. For each filtering method, a figure consisting of original, noised, filtered and difference image was prepared. The difference image was calculated as an absolute difference between the original and filtered data and visualizes advantages and disadvantages of each denoising method.

**Figure 5:**
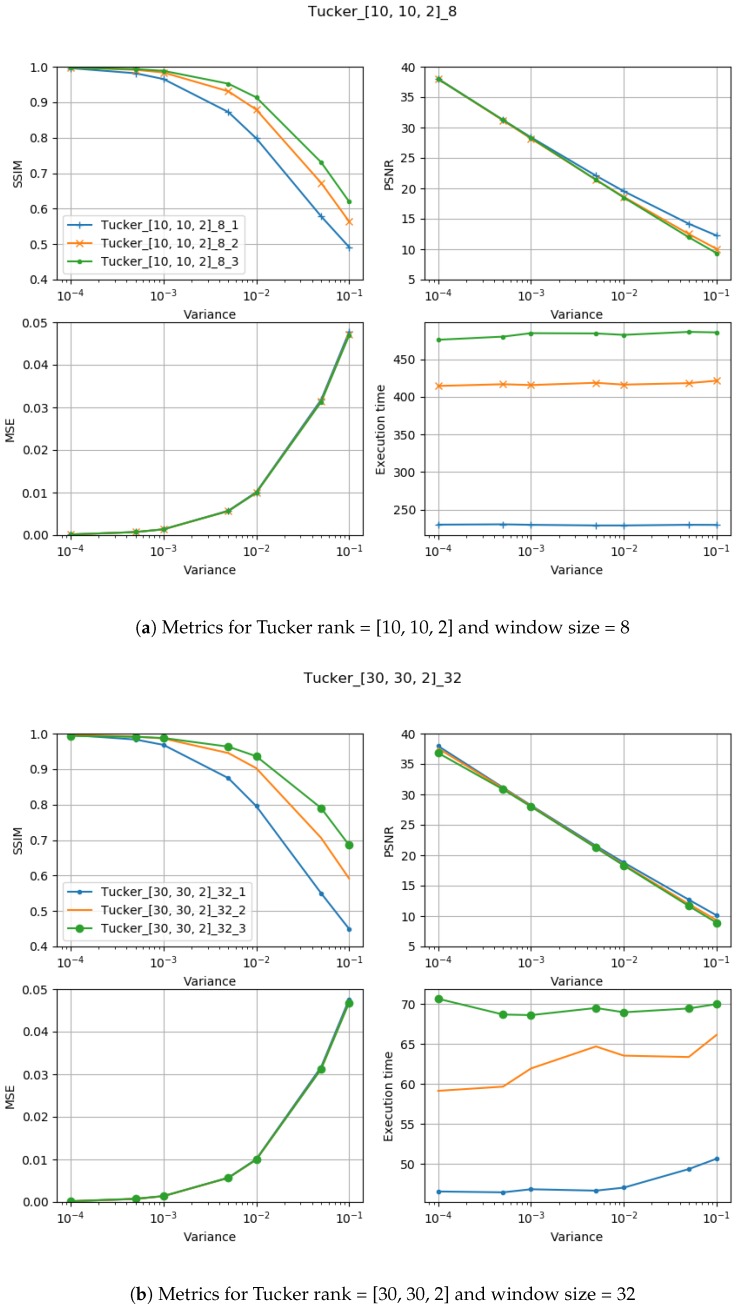

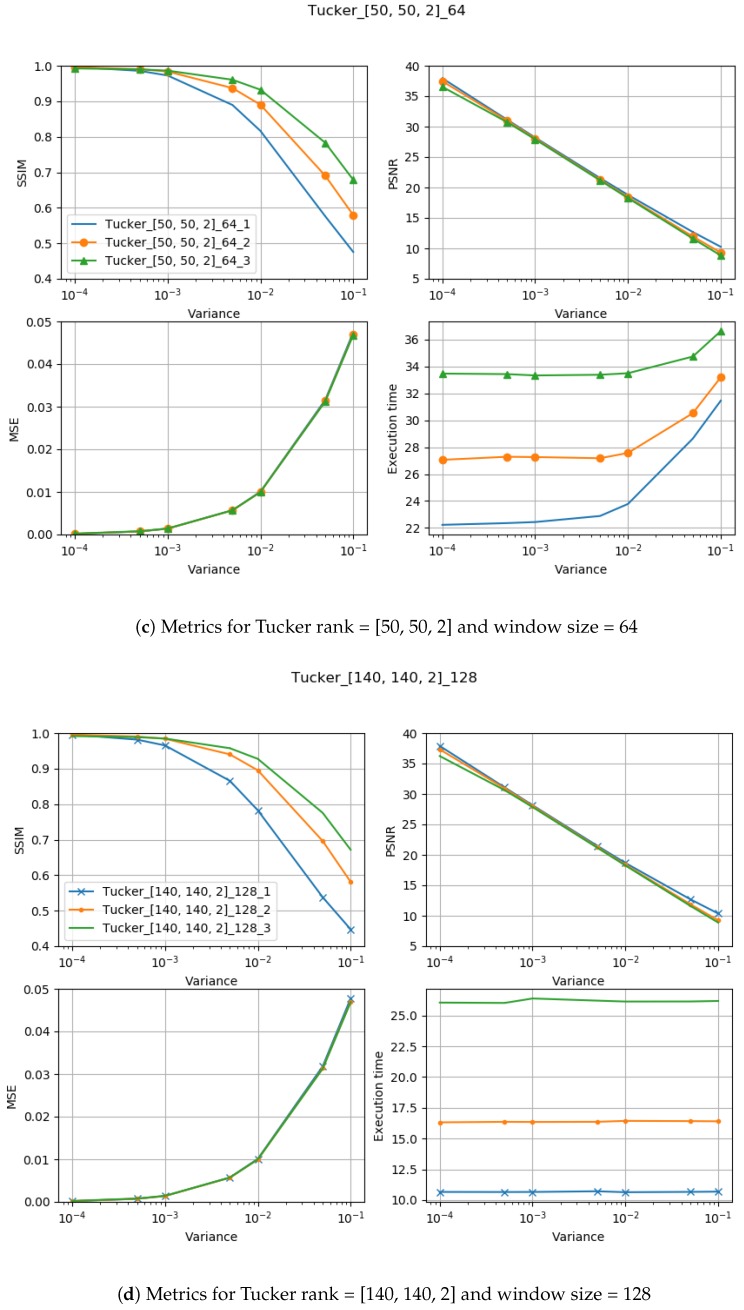
Method performance measured with MSE and SSIM metrics in respect to the noise variance $\sigma^{2}$ as defined in Equation (18).

There are few visible trends between changes in the input parameters and achieved values of the MSE, PSNR, and SSIM, as well as in respect to the execution time. The resulting figures with plotted metrics against noise variance are presented in Figure 5.

**Figure 6:**
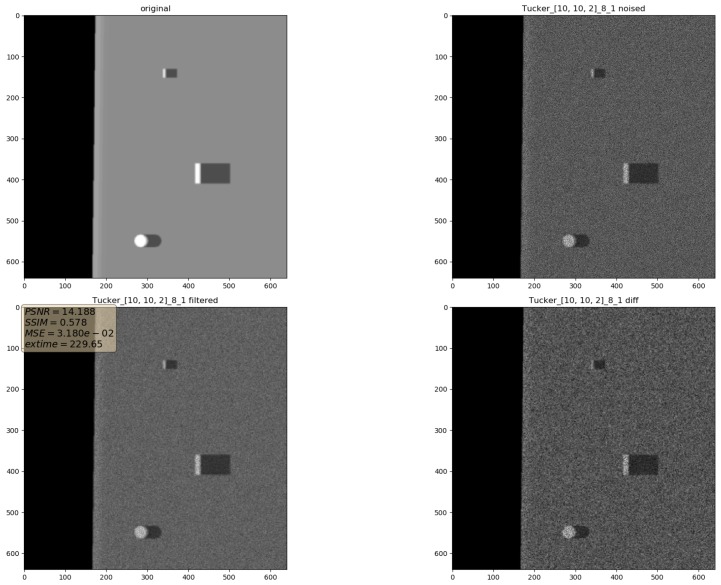
Filtering results for parameters: Tucker rank = [10, 10, 2], window size = 8, close neighbor distance = 1. Speckle noise $\sigma^{2}=0.05$.

**Figure 7:**
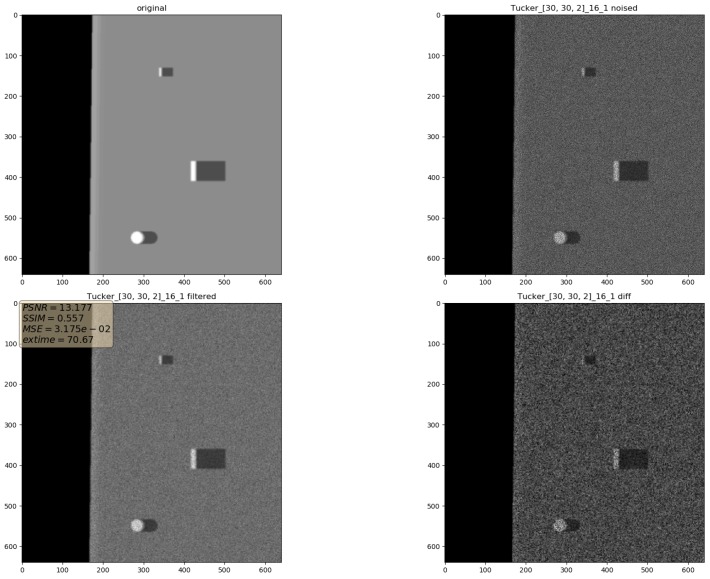
Filtering results for parameters: Tucker rank = [30, 30, 2], window size = 16, close neighbor distance = 1. Speckle noise $\sigma^{2}=0.05$.

**Figure 8:**
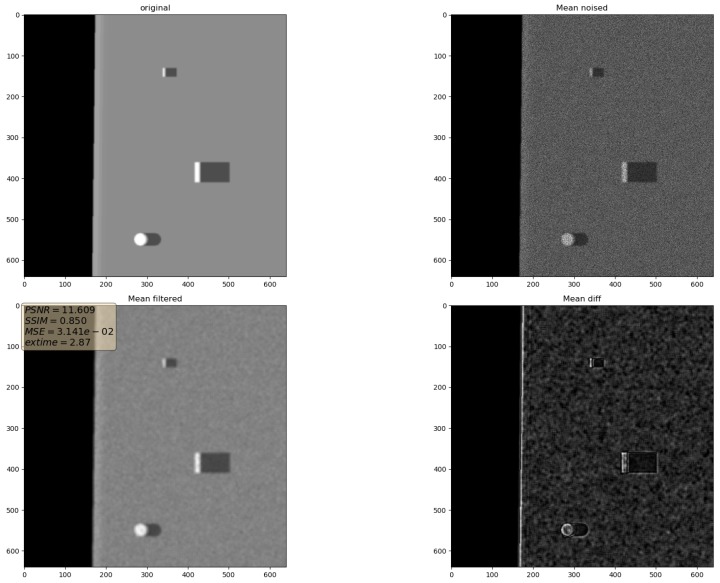
Filtering results for Mean filter. Speckle noise $\sigma^{2}=0.05$.

**Figure 9:**
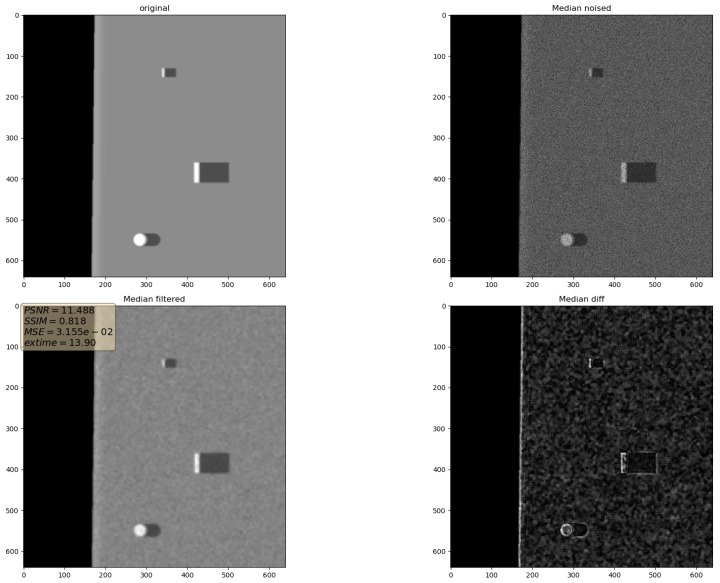
Filtering results for Median filter. Speckle noise $\sigma^{2}=0.05$.

**Figure 10:**
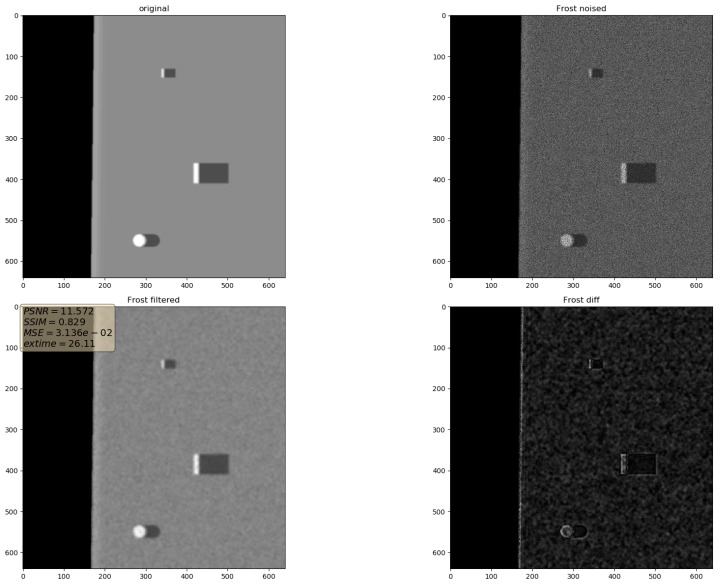
Filtering results for Frost filter. Speckle noise $\sigma^{2}=0.05$.

**Figure 11:**
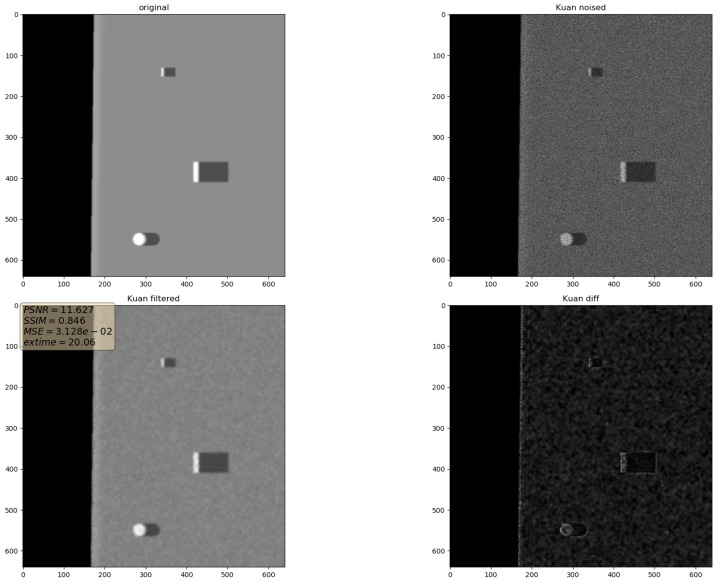
Filtering results for Kuan filter. Speckle noise $\sigma^{2}=0.05$.

**Figure 12:**
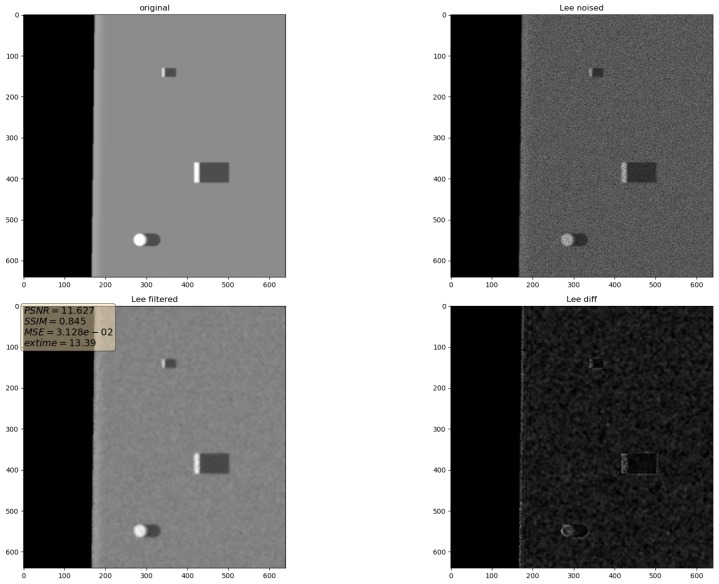
Filtering results for Lee filter. Speckle noise $\sigma^{2}=0.05$.

**Figure 13:**
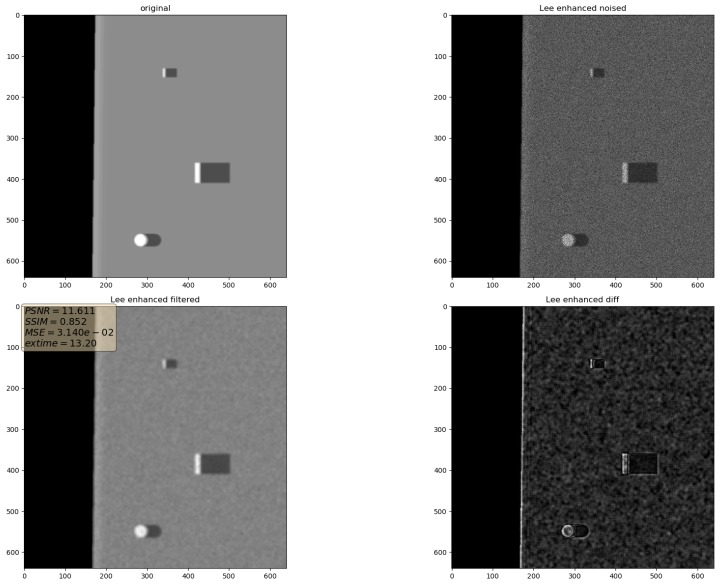
Filtering results for Lee enhanced filter. Speckle noise $\sigma^{2}=0.05$.

It can be observed that increasing the CND parameter improves the SSIM metrics due to better averaging capabilities of the proposed filter with a larger dataset. The improvement is more significant for higher noise variance values. At the same time, a bigger value of this parameter increases the execution time and slightly worsens the PSNR. On the other hand, decreasing rank values of the Tucker decomposition reduces execution time and does not affect significantly achieved metrics values. As found experimentally, the filters achieve their best SSIM performance with window size =32, 64 and window size =8 for the PSNR, respectively. Performance of the methods for which their maximal value in the Tucker rank is lower than the window size, is correlated with the execution time and the noise variance level. Such correlation is not observed for filters with maximal value in the Tucker rank greater than the window size, however. The achieved values of the SSIM metrics for the proposed method usually are lower in the case of the selected benchmark methods. The difference is more significant for higher noise variance, which leads to a conclusion that the proposed method has lower filtering capabilities of structural distortions. The greater impact of structural component is visible in Figure 6 and Figure 7. However, Figure 8, Figure 9, Figure 10, Figure 11, Figure 12 and Figure 13 show higher edge degradation in the benchmark methods.

**Figure 14:**
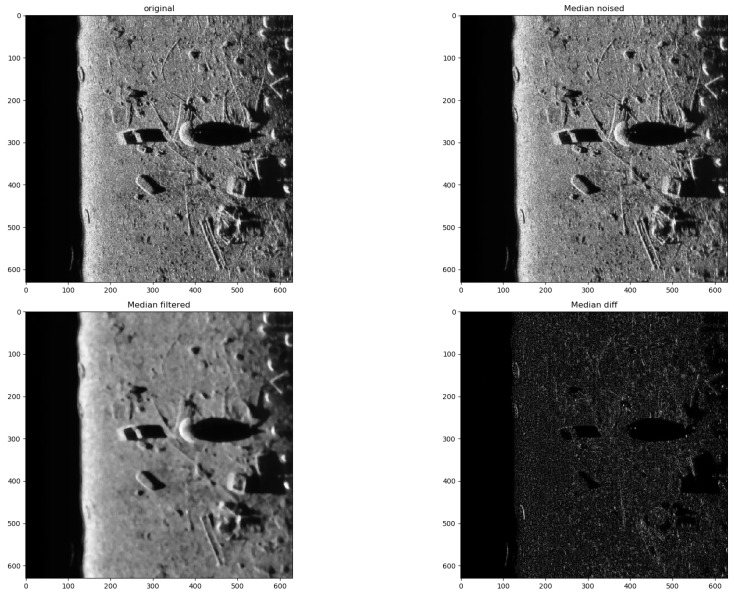
Filtering results for Median filter.

**Figure 15:**
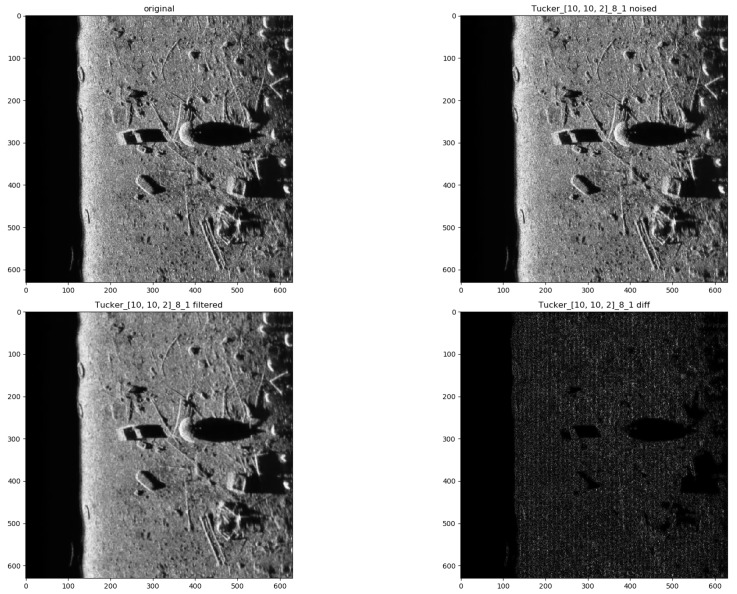
Filtering results for parameters: Tucker rank = [10, 10, 2], window size = 8, close neighbor distance = 1.

**Figure 16:**
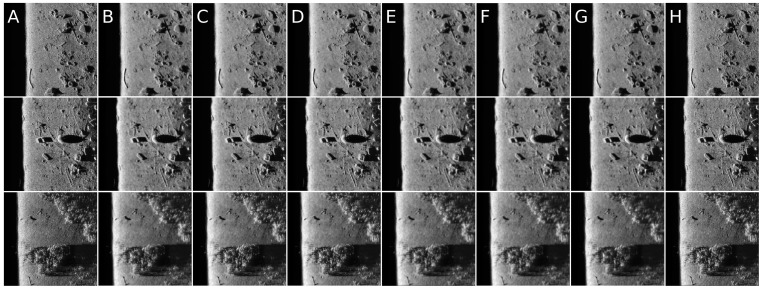
Filtering results comparison for three different real side scan sonar images: (**A**) Original image; (**B**) Frost; (**C**) Kuan; (**D**) Lee; (**E**) Lee enhanced; (**F**) Mean; (**G**) Median; and (**H**) PAuto.

Hence, the proposed method has better edge preservation capabilities than benchmark methods, at least as measured in the available test signals. Comparison between Figure 14 and Figure 15 shows significantly less edge erosion for presented method. The comparison of results in real side-scan sonar images are presented in Figure 16.

## 6. Conclusions

In this paper, a novel filtering method for speckle noise removal in sonar images is presented. The method is based on the Tucker decomposition of tensors composed of local patches of the sonar images. The presented algorithm performs well against images corrupted with speckle noise characteristic of the broad variance range. It outperformed all used benchmark methods in terms of the PSNR and MSE measures. As experimentally observed, the second advantage of the proposed method is less degradation of the important features, such as edges. However, a disadvantage is the high computational cost, especially observed for smaller sized patches. On the other hand, the Python implementation leaves space for further improvements in this respect. Although the method was developed mainly for sonar image enhancement, it can be useful for other signals contaminated with speckle noise such as ultrasound, radar or the OCT images.

## Figures and Tables

**Table 1:** Results of speckle noise reduction for different algorithms. The best results of each case among these methods are denoted by boldface.

$\sigma^{2}$	Index	Methods								
		Mean	Median	Frost	Lee	Lee Enhanced	Kuan	P1	P2	PAuto
0.0001	SSIM	0.98618	0.99462	0.99059	0.98967	0.98871	0.98964	0.99223	**0.99595**	0.9885
	PSNR	33.08012	34.28427	33.80562	33.9245	33.23504	33.89692	34.66237	35.536	**35.59227**
	MSE	4.801 × 10${}^{-4}$	3.701 × 10${}^{-4}$	4.165 × 10${}^{-4}$	4.052 × 10${}^{-4}$	4.655 × 10${}^{-4}$	4.074 × 10${}^{-4}$	3.496 × 10${}^{-4}$	**2.932** × 10${}^{-4}$	2.96 × 10${}^{-4}$
0.0005	SSIM	0.9832	**0.99067**	0.98762	0.98663	0.98567	0.98661	0.98882	0.98155	0.96107
	PSNR	27.7841	28.15245	28.01909	28.06141	27.83237	28.05204	28.42009	28.75497	**28.86646**
	MSE	1.550 × 10${}^{-3}$	1.435 × 10${}^{-3}$	1.478 × 10${}^{-3}$	1.469 × 10${}^{-3}$	1.536 × 10${}^{-3}$	1.472 × 10${}^{-3}$	1.381 × 10${}^{-3}$	**1.335** × 10${}^{-3}$	1.348 × 10${}^{-3}$
0.001	SSIM	0.97971	**0.98621**	0.98424	0.98303	0.98208	0.98303	0.9847	0.96531	0.93043
	PSNR	25.1087	25.32174	25.24272	25.27095	25.1348	25.26519	25.54454	25.89716	**26.05117**
	MSE	2.723 × 10${}^{-3}$	2.613 × 10${}^{-3}$	2.649 × 10${}^{-3}$	2.638 × 10${}^{-3}$	2.710 × 10${}^{-3}$	2.641 × 10${}^{-3}$	2.533 × 10${}^{-3}$	**2.499** × 10${}^{-3}$	2.527 × 10${}^{-3}$
0.005	SSIM	0.95713	0.95753	**0.9612**	0.96007	0.9592	0.96011	0.95523	0.87239	0.83173
	PSNR	18.51647	18.58405	18.55519	18.56429	18.52239	18.56241	18.74906	19.55913	**19.675**
	MSE	9.949 × 10${}^{-3}$	9.900 × 10${}^{-3}$	9.876 × 10${}^{-3}$	9.854 × 10${}^{-3}$	9.938 × 10${}^{-3}$	9.858 × 10${}^{-3}$	**9.704** × 10${}^{-3}$	9.746 × 10${}^{-3}$	9.787 × 10${}^{-3}$
0.01	SSIM	0.93345	0.92814	**0.93689**	0.93593	0.93518	0.93601	0.92394	0.79781	0.74624
	PSNR	15.61236	15.65576	15.63202	15.64155	15.61553	15.64034	15.85178	16.95285	**17.12022**
	MSE	1.670 × 10${}^{-2}$	1.672 × 10${}^{-2}$	1.663 × 10${}^{-2}$	1.660 × 10${}^{-2}$	1.669 × 10${}^{-2}$	1.661 × 10${}^{-2}$	**1.644** × 10${}^{-2}$	1.655 × 10${}^{-2}$	1.664 × 10${}^{-2}$
0.05	SSIM	0.80955	0.78359	0.79237	0.80542	**0.81024**	0.80592	0.76942	0.57621	0.64057
	PSNR	9.02068	9.11328	9.04919	9.16097	9.02168	9.12861	9.43093	**11.63039**	11.47503
	MSE	4.618 × 10${}^{-2}$	4.643 × 10${}^{-2}$	4.616 × 10${}^{-2}$	4.607 × 10${}^{-2}$	4.617 × 10${}^{-2}$	4.608 × 10${}^{-2}$	**4.591** × 10${}^{-2}$	4.635 × 10${}^{-2}$	4.605 × 10${}^{-2}$
0.1	SSIM	0.71967	0.68468	0.63478	0.48404	**0.71972**	0.49222	0.66803	0.48759	0.54534
	PSNR	6.29977	6.40971	6.45613	9.15216	6.3006	8.96839	6.88114	**9.69316**	9.4846
	MSE	6.521 × 10${}^{-2}$	6.557 × 10${}^{-2}$	6.529 × 10${}^{-2}$	6.579 × 10${}^{-2}$	6.520 × 10${}^{-2}$	6.571 × 10${}^{-2}$	**6.496** × 10${}^{-2}$	6.561 × 10${}^{-2}$	6.520 × 10${}^{-2}$

**Table 2:** Input parameters for the P1 and P2 methods.

Parameter	P1 Value	P2 Value
Tucker rank	30, 30, 2	10, 10, 2
Window size	32	8
Close neighbor distance (CND)	3	1

**Table 3:** Average execution time.

Method	Mean	Median	Frost	Lee	Lee Enhanced	Kuan	P1	P2	PAuto
Average time [s]	2.86	13.83	26.06	13.42	13.29	20.12	69.44	482.34	448.52
